# Coronary microvascular disease as potential mechanism of coronary epicardial atherogenesis: an overview of current evidence

**DOI:** 10.3389/fcvm.2026.1946683

**Published:** 2026-10-22

**Authors:** Elio Salameh, Malak Hoballah, Elnaz Ebrahimihoor, Armin Zadeh, Joanne D. Schuijf, Joao A. C. Lima

**Affiliations:** 1 Division of Cardiology, Department of Medicine, Johns Hopkins University, Baltimore, MD, United States; 2 Department of Medicine, University of Pennsylvania, Philadelphia, PA, United States; 3 Global RDC, Canon Medical Systems Europe, Amstelveen, Netherlands

**Keywords:** cardiac imaging, cardiovascular disease, coronary artery disease, coronary microvascular disease, heart failure with preserved ejection fraction

## Abstract

Coronary artery disease (CAD) remains a leading global health burden despite major advances in prevention and treatment, highlighting limitations of the traditional epicardial plaque-centric framework. Increasing evidence suggests that myocardial coronary microvascular disease (CMD) may represent an upstream determinant of atherogenesis. This review synthesizes experimental, clinical, and imaging data supporting the hypothesis that microvascular dysfunction may contribute to the initiation and progression of epicardial atherosclerosis. The latter may, in turn, contribute to CMD, and the relationship between the two compartments is best understood as bidirectional rather than strictly unidirectional. Mechanistically, microvascular dysfunction increases coronary resistance and impairs distal runoff, leading to alterations in wall shear stress that may promote endothelial dysfunction, lipid infiltration, and plaque development in upstream epicardial vessels. Experimental models demonstrate that microvascular abnormalities often precede overt macrovascular disease, while multimodality imaging studies consistently show impaired myocardial perfusion and reduced coronary flow reserve in the absence of obstructive CAD. These functional abnormalities are strongly associated with adverse cardiovascular outcomes, including major adverse cardiovascular events (MACE) and heart failure with preserved ejection fraction (HFpEF). The frequent discordance between perfusion deficits and epicardial stenosis further supports a central role for microvascular dysfunction in ischemic heart disease. Collectively, these findings challenge a purely anatomical framework and highlight the value of quantitative imaging of coronary flow and perfusion for disease characterization and risk stratification. A shift toward a microvascular-centered paradigm may enable earlier detection, improve phenotyping, and inform targeted preventive and therapeutic strategies.

## 1 Introduction: a paradigm shift

Despite the remarkable gains in primary and secondary prevention, coronary artery disease (CAD) and vascular atherosclerosis continue to increase in prevalence and burden worldwide, due to the rise of obesity and type II diabetes mellitus (T2DM), leading to the modern cardiometabolic disease epidemics (1, 2). Over the past three decades, this epidemiologic transition has reshaped cardiovascular diseases (CVD), with coronary atherosclerotic manifestations—including heart failure, atrial fibrillation, and stroke—emerging as leading causes of morbidity, cognitive decline, and mortality (3–5). Paradoxically, this growing burden has coincided with transformative advances in cardiovascular imaging which now enable detailed, noninvasive characterization of coronary anatomy, myocardial structure, perfusion, and tissue composition. In parallel, therapeutic strategies targeting atherogenesis (6), hypertension (7), ischemic heart disease (8) and heart failure (9) have been recently augmented by pharmacologic strategies to control metabolic diseases directly such as SGLT2 inhibitors (10) and GLP-1 agonists (11).

Yet, despite these advances, contemporary diagnostic and therapeutic paradigms remain largely anchored in an epicardial plaque-centric view of disease, in which epicardial coronary atherosclerosis represents the primary substrate of pathology. Imaging strategies including coronary computed tomography angiography (CCTA), invasive coronary angiography, and intravascular imaging have reinforced this paradigm by focusing on detection and characterization of epicardial plaques, with prophylactic efforts mainly targeting the prevention of macrovascular disease. In contrast, coronary microcirculation has historically been under-recognized, often viewed as a secondary or parallel process rather than a central driver of disease.

This review proposes an alternative, imaging-informed paradigm: that myocardial microvascular disease represents a fundamental and potentially primary substrate of cardiovascular pathology. We posit that CMD alters coronary flow dynamics, reduces distal runoff, and induces adverse wall shear stress (WSS) patterns that may promote epicardial atherogenesis, while simultaneously driving consequences such as ischemia, inflammation and fibrosis. However, the reverse relationship is also plausible, where established epicardial atherosclerosis, diffuse endothelial dysfunction, and shared cardiometabolic risk factors (i.e., diabetes, hypertension, obesity, dyslipidemia, inflammation, oxidative stress) may themselves promote microvascular injury. Imaging techniques such as perfusion imaging, quantitative flow assessment, and tissue characterization by cardiac magnetic resonance (CMR) and positron emission tomography (PET), offer unique opportunities to interrogate microvascular function *in vivo*. This review condenses evidence from basic, clinical and population-based studies that support microvascular-induced coronary atherogenesis as an important contributor to cardiac disease pathophysiology. By integrating these insights within an imaging framework, we highlight the potential for microvascular phenotyping to redefine risk stratification, enable earlier disease detection, and inform the development of targeted therapeutic strategies.

This review differs from prior syntheses of CMD in that it focuses specifically on the evidence that CMD may act as an upstream contributor to epicardial atherogenesis, rather than categorizing CMD as a parallel or downstream process. It integrates recent imaging-based mechanistic data not covered in earlier reviews, while explicitly weighing the strength and limitations of the underlying evidence base. The key terms used in this article are explained in Box 1.

BOX 1 **Terminology.**

Coronary microvascular dysfunction (CMD): functional and/or structural abnormalities of the coronary microcirculation (vessels <500 μm) resulting in impaired vasodilator capacity, increased microvascular resistance, microvascular spasm, or other abnormalities of microvascular function, with or without symptoms. Ischemia with no obstructive coronary artery disease (INOCA): a clinical syndrome characterized by angina and/or objective myocardial ischemia in the absence of obstructive epicardial coronary artery disease (typically <50% stenosis); CMD is one, but not the only, underlying mechanism of INOCA. Microvascular angina: anginal symptoms and/or myocardial ischemia attributable to demonstrated coronary microvascular dysfunction; a clinical endotype of INOCA in which CMD has been identified as the likely mechanism. Nonobstructive coronary artery disease: an anatomic/angiographic description denoting the absence of obstructive epicardial stenosis (typically <50%); it does not, by itself, imply normal coronary microvascular or vasomotor function. These terms are used according to the above definitions throughout this review.

## 2 Mechanistic framework: from microvascular dysfunction to epicardial atherogenesis

### 2.1 Revisiting atherogenesis through a hemodynamic lens

Atherogenesis has been the focus of extensive fundamental, clinical, and population research. Our current understanding encompasses its underlying mechanisms (12), the tempo of sequential activation and halt (12), the natural history of atherosclerosis (13), and the genetic and environmental factors (14) that predispose individuals at the population level—all of which have been previously described and discussed in detail. The prevailing model emphasizes lipid infiltration through intercellular space between endothelial cells (13), inflammation with macrophage infiltration, and plaque development within the epicardial arterial wall (13), culminating in rupture, thrombosis, and clinical events (12, 15–17).

However, this framework incompletely accounts for the hemodynamic forces governing plaque initiation and distribution, particularly wall shear stress (WSS), which serves as a critical link between flow and vascular biology.

### 2.2 Wall shear stress and atherogenesis

Arterial wall shear stress (WSS) represents the tangential force created by blood flow against the arterial intima (18), and is thought to regulate arterial growth through biologic processes that include nitric oxide synthesis and degradation (19). Endothelial surfaces exposed to low WSS have greater endothelial intercellular spaces through which lipidic rich particles can more easily penetrate the arterial wall, increasing predisposition to atherogenesis (20). Therefore, local alterations in WSS in large part define arterial plaque distribution, particularly at arterial bends, tortuosity, tributary bifurcation and intra-myocardial bridges (21, 22).

The relationship between WSS and atherosclerosis has been extensively demonstrated across multiple vascular territories, including the aorta (23), carotid arteries (18) and other large thoracic, abdominal and cerebral arterial conduits (24, 25). Early assessments relied on computational flow dynamics, while subsequent investigations incorporated doppler and magnetic resonance-based technologies to assess shear stress *in vivo* (26). More recent methods based on tracking minute differences in arterial flow in large vessels have used MRI 4D-5D flow techniques but have not been consistently applied to coronaries due to intrinsic limitations in spatial resolution (27, 28). In the coronary circulation, WSS mapping using computational models has demonstrated a strong association between low WSS and plaque initiation and progression (29), although clinical implementation has primarily focused on estimating pressure drops across potentially obstructive lesions rather than characterizing upstream disease mechanisms (30, 31).

A critical determinant of WSS is the magnitude and pattern of blood flow. Indeed, the concept that flow disturbances predispose to atherosclerosis was originally proposed by Leonardo Da Vinci centuries ago (19). The interplay of alterations of WSS, tensile stress and arterial wall biology have been studied in human coronary arteries and the notions that flow reduction and departure from undisturbed laminar flow profiles predispose to atherosclerosis have been known for decades (21).

Clinically, some of the most compelling examples of flow reduction as the main factor underlying aggressive atherogenesis and plaque calcification comes from clinical observations in patients treated with coronary artery bypass grafting (CABG) for revascularization of advanced CAD (32). The accelerated progression of atherosclerosis in arterial segments proximal to the bypass anastomoses are thought to result from reduced flow given competition with flow from the bypass conduit, including the left internal mammary artery, saphenous vein grafts, or other arterial or venous conduits used to treat the upstream coronary obstructive stenosis(es) (32, 33). In segments where the bypass graft involutes given high flow in the native artery, such accelerated progression is not seen.

### 2.3 Determinants of myocardial microvascular dysfunction and hemodynamic consequences

Myocardial microvascular dysfunction arises from a convergence of inflammatory, metabolic, and hemodynamic insults that lead to structural and functional alterations in the microcirculation. The substrate for microvessel wall thickening and occlusion has been attributed to chronic inflammation (34), hyperglycemia-mediated endothelial damage (35), and smooth muscle cell hyperplasia in response to increased intravascular pressure (36). The frequent coexistence with macrovascular atherosclerosis has led to speculation on the possible role of hypercholesterolemia and increased serum triglyceride levels (37).

Additional hemodynamic factors further exacerbate microvascular injury. Elevated intra-cavitary pressures, as seen in cardiomyopathies, may compress the microvasculature, contributing to its dysfunction and associated ventricular damage (38). The presence of peri-vascular fibrosis is frequent and may represent a consequence and/or cause of myocardial microvessel dysfunction (39). Myocardial inflammation, ubiquitous across most cardiac pathologies, represents a common denominator in the pathogenesis of microvascular disease (40). It encompasses not only primary cardiac inflammatory processes—such as myocarditis, myocardial vasculitis, and pericarditis—but also systemic causes of myocardial inflammation that secondarily affect the heart such as obesity, dysmetabolism and diabetes mellitus. These processes promote endothelial dysfunction, oxidative stress, and structural remodeling of the microvasculature. In this regard, advances in obesity pharmacotherapy, particularly the GLP-1 receptor agonists like semaglutide, have been shown to attenuate cardiac microvascular injury in diabetes-induced mice by preserving microvascular structure and density and inhibiting perivascular fibrosis (41). This is possible due to semaglutide's activation of antioxidant and inhibition of pro-inflammatory pathways, both central to maintaining microvascular integrity (42).

Functionally, CMD leads to impaired myocardial perfusion and ischemia, initiating cycles of tissue injury and repair processes that perpetuate local inflammatory signaling, ultimately resulting in secondary interstitial or replacement fibrosis (43, 44). These changes contribute to ventricular remodeling, including reactive hypertrophy, reduced compliance, impaired relaxation, and ultimately systolic and/or diastolic dysfunction (45, 46). Microvascular pathology is therefore a common mechanism of cardiac disease and dysfunction even when unassociated with macrovascular pathology. However, frequently also, the two processes overlap.

### 2.4 Myocardial microvascular disease as an upstream driver of atherogenesis

CMD has long been recognized in association with hypertension, diabetes mellitus, kidney disease, and cardiomyopathies (47–50), and is a well established contributor to ischemia, particularly in patients with ischemia and nonobstructive coronary arteries (INOCA) (51). However, CMD is typically conceptualized as a disease process occurring in parallel with macrovascular atherosclerosis rather than as a contributor to its development.

A critical but underappreciated determinant of epicardial coronary flow is the integrity of the downstream microcirculation. In fact, microvascular resistance is the main determinant of coronary blood flow (52), with coronary microcirculation accounting for at least 60% of total coronary vascular resistance (53). An increased microvascular resistance creates a reduction in resting coronary blood flow and particularly its response to increased demand, which significantly affects endothelial shear stress upstream throughout the coronary vasculature. This relationship has been directly demonstrated: coronary segments in arteries with abnormal microvascular function exhibit significantly lower endothelial shear stress (ESS) compared with segments in arteries with normal microvascular function (average ESS: 1.67 ± 1.04 Pa vs. 2.03 ± 1.72 Pa, *p* = 0.050; lowest ESS: 0.54 ± 0.25 Pa vs. 0.72 ± 0.32 Pa, *p* = 0.014) (54). Additionally, all patients with epicardial endothelial dysfunction also had microvascular endothelial dysfunction, suggesting that the low epicardial shear stress associated with CMD may act as a stimulus for epicardial endothelial dysfunction and/or vice versa. Low wall shear stress itself promotes endothelial dysfunction through multiple pathways, including downregulation of nitric oxide synthase, increased inflammatory gene expression, and transformation of endothelial cells to a proatherogenic phenotype (55). Recent evidence demonstrates that microvascular resistance remains largely stable and independent of epicardial resistance changes (56). When epicardial stenosis severity increases, epicardial resistance rises substantially while hyperemic microvascular resistance remains constant (56). Under physiological conditions, coronary autoregulation buffers moderate increases in microvascular resistance, maintaining relatively stable resting myocardial blood flow across a range of perfusion pressures and allowing epicardial flow and WSS to remain largely preserved (52). This independence confirms that microvascular dysfunction represents a distinct pathophysiological process that alters flow dynamics through its own resistance properties rather than as a secondary consequence of epicardial disease. The hemodynamic consequences of microvascular dysfunction on upstream epicardial flow are therefore expected to become most apparent once autoregulatory reserve is exhausted. Examples include states of increased myocardial oxygen demand, or when microvascular resistance is severely and diffusely elevated, as in diabetes, hypertension, and INOCA (47, 57, 58).

Endothelial dysfunction and reduced nitric oxide bioavailability also underlie vasospastic angina. Vasospasm occurs disproportionately at sites of myocardial bridging and at segments harboring small, nonobstructive atherosclerotic plaques, where impaired endothelium-dependent vasodilation and heightened smooth muscle reactivity coexist (59). In myocardial bridging, systolic compression of the tunneled arterial segment is thought to injure the adjacent endothelium and reduce local nitric oxide synthesis, while the proximal segment—exposed to disturbed, low-shear flow—is prone to both endothelial dysfunction and atherosclerotic plaque formation, providing a shared substrate for vasospasm and atherogenesis at the same anatomic location. This overlap reinforces the concept that endothelial and microvascular dysfunction, rather than a single unifying anatomic lesion, may underlie a spectrum of ischemic presentations extending from vasospastic angina to epicardial atherogenesis.

Taken together, this framework supports a shift from viewing microvascular disease as a downstream consequence to recognizing it as a potential upstream promotor of atherogenesis. By impairing coronary runoff and altering WSS, myocardial microvascular dysfunction may create the hemodynamic conditions inducive to plaque formation, providing a unifying link between microvascular pathology and epicardial CAD.

Figure 1 summarizes the proposed pathway by which myocardial microvascular disease promotes epicardial coronary atherogenesis through impaired coronary runoff, reduced wall shear stress, and endothelial dysfunction.

**Figure 1:**
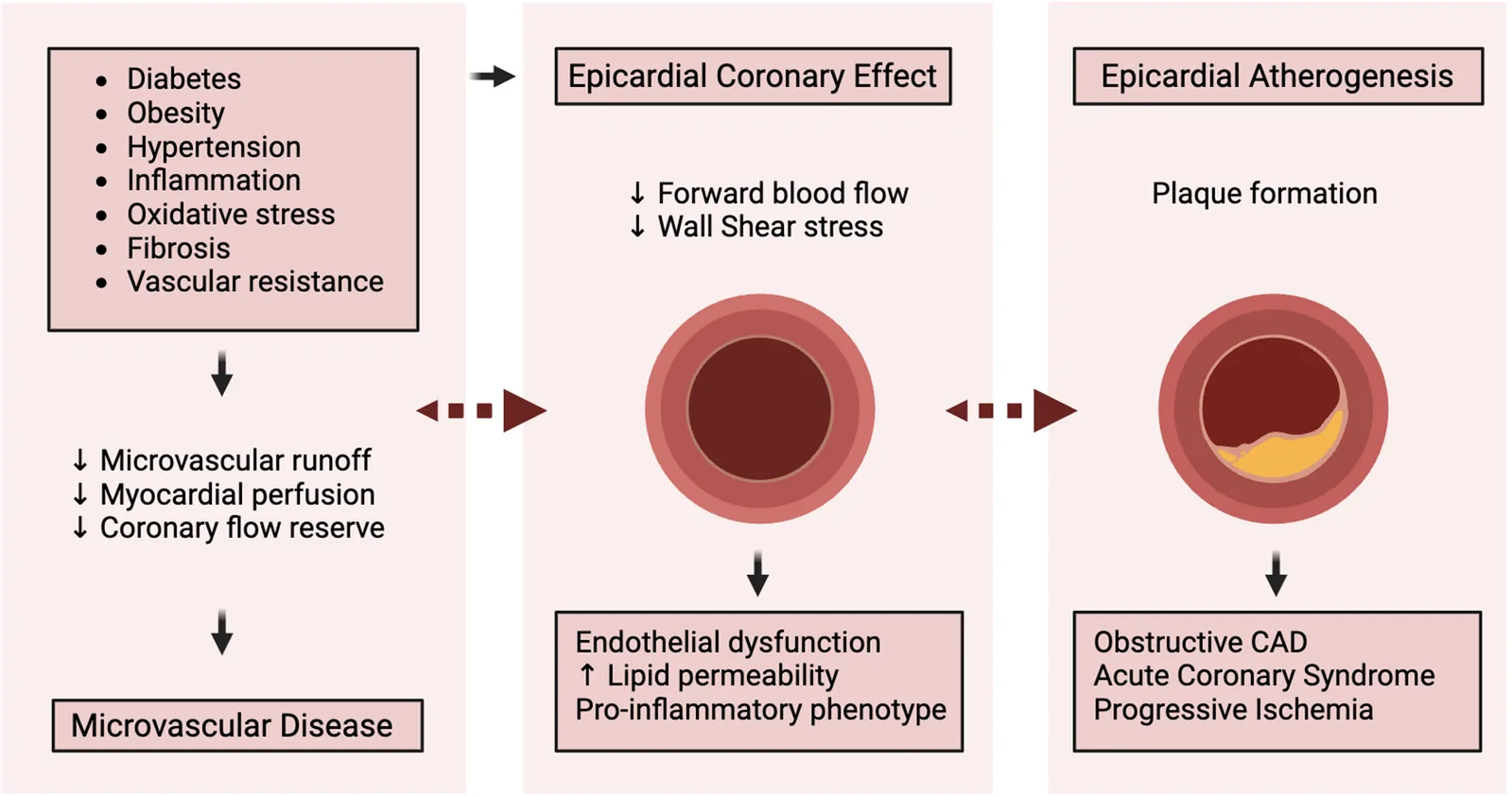
Proposed pathway linking myocardial microvascular disease to epicardial coronary atherogenesis. Dashed bidirectional arrows depict the reciprocal relationship between microvascular and epicardial coronary disease, with the larger arrow representing the proposed pathway and the smaller arrow representing the reverse pathway. Shared upstream cardiometabolic risk factors (left panel) contribute to disease in both vascular compartments, reflecting their common pathobiologic drivers. This schematic illustrates a proposed conceptual framework and does not imply an established causal sequence.

## 3 Evidence supporting a microvascular-to-macrovascular continuum

### 3.1 Experimental evidence: temporal primacy of microvascular dysfunction

Experimental models have provided key insights into the temporal relationship between microvascular dysfunction and epicardial atherogenesis. In swine, exposure to hypercholesterolemia with or without hyperglycemia, induces early microvascular dysfunction characterized by impaired endothelium-dependent vasorelaxation and increased microvascular passive resistance in the absence of epicardial plaque within 10 to 20 weeks (60). With prolonged exposure (∼15 months), both microvascular and macrovascular structural changes emerge, although observed epicardial atherosclerotic lesions remained non-flow limiting (plaque burden <30%) (61). Similarly, combined cardiometabolic stressors (hypertension, diabetes, hypercholesterolemia) result in sustained levels of inflammation and oxidative stress, reduced capillary density, increased myocardial fibrosis with elevated cardiomyocyte stiffness, and diastolic dysfunction, highlighting the early myocardial consequences of microvascular injury (62). Accordingly, these animal models provide insights on how exposure to risk factors may trigger pathophysiological changes in the microvasculature at first, followed by overt changes in the macrovasculature and cardiac function over time.

Complementary evidence from a hamster model demonstrates that diabetes and hyperlipidemia synergistically accelerate both micro- and macrovascular pathology, with microvascular alterations (capillary narrowing, basement membrane thickening, perivascular matrix expansion) preceding the development of overt macrovascular atherosclerosis (63). Notably, diabetes alone produced similar microvascular and macrovascular alterations, but at a slower pace, whereas hyperlipidemia alone induced only macrovascular atherosclerotic changes without affecting the microvasculature. These findings indicate that diabetes-related microvascular injury may represent an early pathological event that can arise independently of, and before, the development of overt macrovascular atherosclerosis.

Table 1 summarizes key experimental studies highlighting the progression from myocardial microvascular disease to epicardial plaque formation.

**Table 1:** Key experimental studies showing the progression from microvascular to macrovascular coronary disease.

Study	Model	Duration	Key Findings	Importance	References
Van den Heuvel, 2012	Swine (DM + HFD)	10–20 weeks	↓ NO-mediated vasodilation; ↓ ET-1 responses; no epicardial plaque	Early CMD precedes plaque formation	(60)
Sorop, 2016	Swine (DM + HFD)	15 months	Non flow-limiting plaques; ↑ ET-1 vasoconstriction; ↑ vascular stiffness	Time-dependent progression: functional → structural changes	(61)
Sorop, 2018	Swine (DM + HFD + HTN)	6 months	↑ Myocardial collagen; ↓ capillary/fiber ratio; ↑ cardiomyocyte stiffness; LV diastolic dysfunction	Multiple comorbidities recapitulate HFpEF pathophysiology	(62)
Simionescu, 1996	Hamster (DM ± HL)	2–24 weeks	DM + HL: concurrent micro/macro-angiopathy; DM alone: slower progression; HL alone: macrovascular only	DM-induced microvascular injury precedes and is independent of macrovascular disease	(63)

DM, diabetes mellitus; HFD, high-fat diet; NO, nitric oxide; ET-1, endothelin-1; CMD, coronary microvascular disease; HTN, hypertension; LV, left ventricular; HFpEF, heart failure with preserved ejection fraction; HL, hyperlipidemia.

Mechanistically, microvascular dysfunction may promote atherogenesis through several pathways: (1) impaired perfusion of the vessel wall through dysfunction of the adventitial vasa vasorum, potentially leading to localized hypoxia that triggers inflammatory responses and neovascularization (32); (2) systemic inflammatory amplification driven by the extensive endothelial surface area of the microcirculation (64); and (3) oxidative stress and endothelial barrier disruption—potentially involving redox-regulatory mechanisms (65)—facilitating lipid infiltration and recruitment of inflammatory cells across both microvascular and macrovascular beds (32).

In apolipoprotein E-deficient mice, hyperglycemia impairs neovascularization of the vasa vasorum and accelerates atherosclerotic development, supporting a direct mechanistic link between microvascular alterations and epicardial plaque progression (66). Taken together, these findings support a bidirectional relationship in which cardiovascular risk factors concurrently affect both vascular compartments through shared pathophysiological mechanisms, including endothelial dysfunction, oxidative stress, and inflammation, and suggest potential feedback interactions between microvascular and macrovascular disease progression.

### 3.2 Clinical and imaging evidence of microvascular-macrovascular interplay

Consistent with observations in animal models, human studies demonstrate that microvascular dysfunction often precedes or coexists with macrovascular atherosclerosis, particularly in individuals with cardiometabolic risk factors.

The Women's Ischemia Syndrome Evaluation (WISE) Study pioneered the systematic investigation of ischemic heart disease in women without obstructive coronary artery disease (67). It identified coronary microvascular dysfunction in approximately 50% of participants (68) and proved it a key contributor to anginal chest pain despite the absence of significant epicardial coronary obstruction (INOCA) (69, 70). Although initially described in women (67), it is increasingly recognized that men are also frequently affected by INOCA (71, 72).

Multimodality imaging studies have been central to characterizing this relationship. PET, CMR, CT perfusion, and SPECT imaging consistently demonstrate that myocardial perfusion abnormalities frequently occur in the absence of obstructive epicardial disease, reflecting microvascular disease rather than “false-positive” findings.

In the CORE320 study (51), perfusion defects were identified in patients without obstructive CAD, and importantly, regions of ischemia often did not correspond to specific coronary territories, supporting a complex coexistence of microvascular ischemia and epicardial atherosclerosis (51). Multiple other studies have also documented the mismatch between obstructive epicardial CAD and perfusion defects across imaging modalities, with perfusion abnormalities in the absence of obstructive CAD being frequent in clinical practice, particularly among women and obese patients (Figure 2). Seminal work by Viviany R. Taqueti and Marcelo F. Di Carli provides a mechanistic framework for this mismatch, demonstrating that diffuse, nonobstructive epicardial atherosclerosis coexists (70%–80%) with coronary microvascular dysfunction, and that many of these vessels exhibit significant longitudinal pressure gradients despite the absence of focal stenosis. This results in impaired coronary flow and myocardial perfusion, helping explain ischemic symptoms despite nonobstructive findings on angiography or coronary CTA (47). In fact, one of the earliest demonstrations of significant myocardial ischemia in non-stenotic coronaries comes from the work of De Bruyne et al., showing that diffuse coronary atherosclerosis without focal stenosis on angiogram causes a graded, continuous pressure fall along the involved artery (73). Consistent with this, other studies (53, 74) documented that even among individuals with obstructive CAD, noninvasive imaging has shown abnormal perfusion in myocardial regions supplied by vessels without apparent obstructive CAD demonstrated by invasive coronary angiography. Similarly, Kero et al. (75) examined 837 symptomatic patients undergoing both coronary CTA and 15O-water PET myocardial perfusion imaging, using AI-guided quantitative CT to assess stenosis and plaque characteristics. They found that many vessels supplying ischemic territories have relatively low stenosis degree and plaque burden, suggesting that factors beyond epicardial atherosclerosis—such as microvascular occlusion or dysfunction—may contribute to myocardial ischemia.

**Figure 2:**
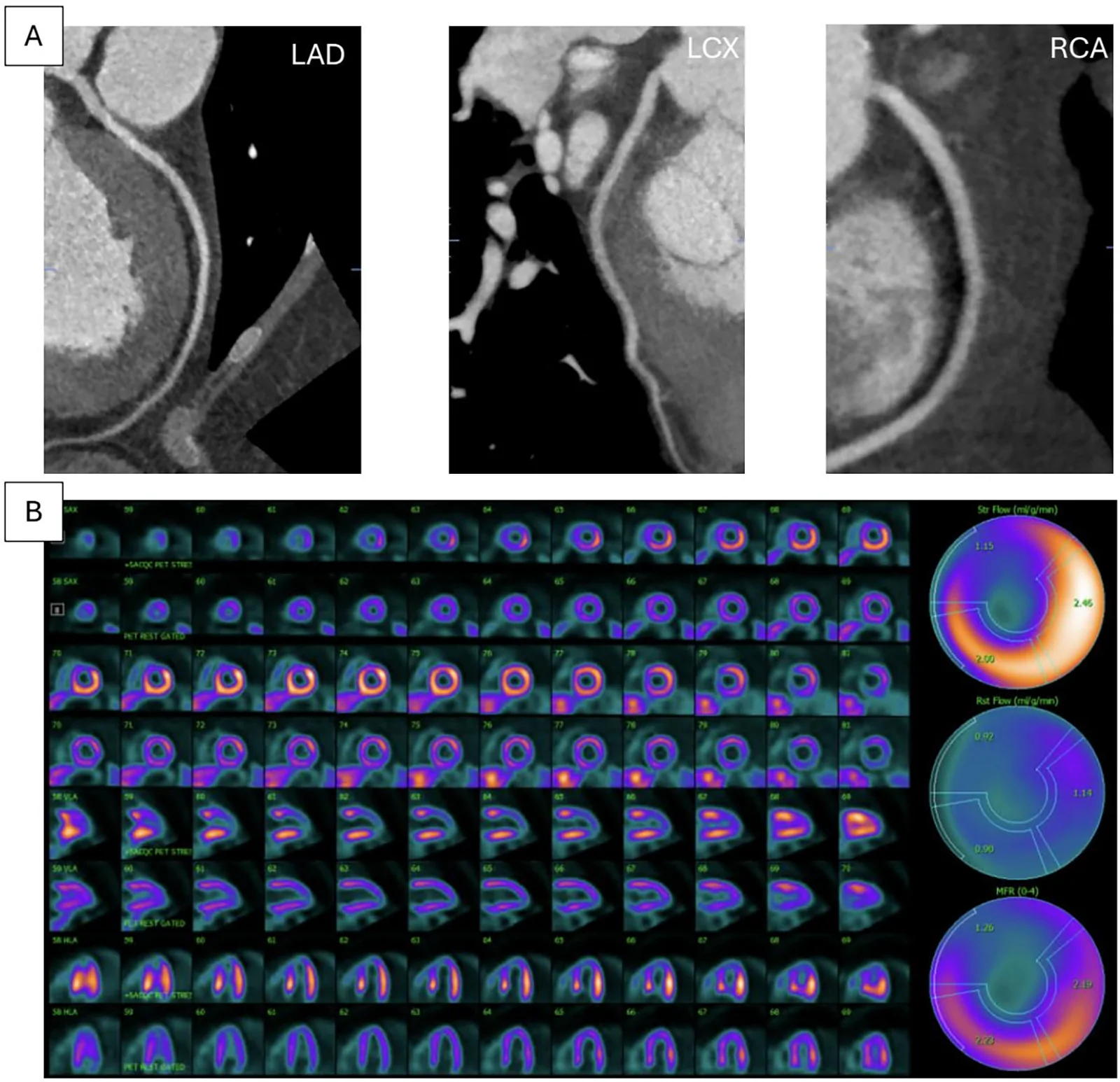
Imaging findings in a 40-year-old woman presenting with anginal symptoms and no obstructive epicardial coronary artery disease, who was eventually diagnosed with coronary microvascular disease. **(A)** Coronary CT angiography curved multiplanar reformations (curved MPR) image of the left anterior descending (LAD), right coronary artery (RCA), and left circumflex (LCX) arteries demonstrating no significant epicardial coronary artery stenosis. **(B)** PET myocardial perfusion imaging demonstrating mild/moderate intensity large area of reversible perfusion abnormality involving the apex and extending into the apical to mid segments of the anterior and anteroseptal wall, with mild extension into the apical inferior wall. No significant rest perfusion abnormalities to suggest prior transmural myocardial infarction. Quantitative analysis of myocardial blood flow demonstrates decreased stress myocardial blood flow and coronary flow reserves in the LAD territory, with preserved myocardial flow reserves in the LCX and RCA territories. This is an original, unpublished clinical image from our own institution's imaging archive, that has been de-identified with no third-party copyright involved.

In the PESA study (Progression of Early Subclinical Atherosclerosis), higher baseline myocardial blood flow (MBF) and myocardial perfusion reserve (MPR)—imaging markers of microvascular function—were associated with reduced progression of atherosclerosis across multiple vascular beds, including coronary, carotid, and femoral territories, as measured by three-dimensional vascular ultrasound and calcium scoring (76). Padro et al, in an ESC Working Group position paper, discussed the evidence showing that coronary microvascular disturbances have been associated with early stages of atherosclerosis even prior to any angiographic evidence of epicardial coronary stenosis (77). These findings support an important prospective association between microvascular dysfunction and macrovascular disease.

Table 2 summarizes the key clinical studies supporting the temporal and mechanistic relationship between microvascular dysfunction and epicardial atherosclerosis.

**Table 2:** Key clinical studies supporting the relationship between microvascular dysfunction and epicardial atherosclerosis progression.

Study	Study design	Population (*n*)	Key findings	Importance	References
PESA (Devesa et al.)	Primary Clinical Study; Prospective cohort; 3-year follow-up	Asymptomatic middle-aged with CV risk factors (*n* = 453)	Higher baseline stress MBF and MPR predicted reduced atherosclerosis progression in coronary, carotid, and femoral arteries	Lower microvascular dysfunction associated with reduced subsequent atherosclerosis progression.	(76)
Siasos et al.	Primary Clinical Study; Cross-sectional with CFD analysis	Early non-obstructive CAD (*n* = 65)	Coronary segments with CMD had lower ESS; all patients with epicardial dysfunction had CMD	Suggestion of a continuum from CMD to epicardial dysfunction through ESS	(54)
Taqueti and Di Carli	Review of Multiple studies	Chest pain, no obstructive CAD, with CMD	70–80% showed diffuse nonobstructive atherosclerosis; >50% had longitudinal pressure gradients	CMD coexists with nonobstructive atherosclerosis	(47)
Del Buono et al.	Review	Multiple populations with CVD	CMD coexists with obstructive CAD and magnifies ischemia; reduced CFR predicts adverse outcomes	CMD coexists with CAD and has independent prognostic value	(53)
Padro et al. (ESC position)	Position paper	Multiple populations	Microvascular disturbances associated with early atherosclerosis prior to angiographic stenosis	CMD may precede epicardial stenosis	(77)
WISE Study	Prospective multicenter cohort study	Women with chest pain and no obstructive CAD (*n* = 936)	CMD identified in ∼50% of participants	Frequent overlap with diffuse nonobstructive atherosclerosis in women	(68)
CORE320 (Schuijf et al.)	Prospective multicenter cohort study	Symptomatic patients referred for angiography (*n* = 381)	8% had INOCA with perfusion defects; perfusion abnormalities often located separate from territories with epicardial stenoses	Perfusion defects exist beyond epicardial stenosis distributions	(51)

CV, cardiovascular; MBF, myocardial blood flow; MPR, myocardial perfusion reserve; CMD, coronary microvascular disease; CFD, computational fluid dynamics; CAD, coronary artery disease; ESS, epicardial shear stress; CVD, cardiovascular disease; CFR, coronary flow reserve; INOCA, ischemia and no obstructive coronary artery disease.

### 3.3 Reverse pathway: epicardial atherosclerosis and shared risk factors as drivers of microvascular dysfunction

While Secs. 3.1 and 3.2 outline evidence for a microvascular-to-epicardial pathway, epicardial atherosclerosis, and the risk factors that drive it, can themselves injure the coronary microcirculation. Mechanistically, the clearest demonstration of this reverse pathway is coronary microembolization: mechanical stress, erosion, or rupture of an epicardial atherosclerotic plaque releases particulate debris and soluble mediators into the coronary circulation; the particulate material physically obstructs the microcirculation, while the soluble mediators induce microvascular endothelial dysfunction and vasoconstriction, together producing patchy microinfarction and impaired microvascular reserve (78, 79). This process occurs both spontaneously, in the setting of plaque erosion or rupture, and periprocedurally, during percutaneous intervention of atherosclerotic lesions, and provides direct mechanistic evidence that epicardial plaque burden and instability can impair microvascular structure and function (80). Consistent with a bidirectional model, diffuse nonobstructive epicardial atherosclerosis has also been associated with more pronounced microvascular remodeling (47), suggesting that the burden of upstream epicardial disease may independently contribute to downstream microvascular impairment. Diffuse, nonobstructive epicardial disease can additionally produce a graded, continuous pressure fall along the length of an artery even without focal stenosis, reducing the driving pressure available to the distal microcirculation and providing a hemodynamic, rather than embolic, route by which epicardial disease burden could impair microvascular perfusion (47). Separately, perivascular fibrosis—a hallmark of microvascular injury—has been proposed to be both a consequence and a driver of microvascular dysfunction (43, 53), underscoring that the two compartments are mechanistically intertwined rather than governed by a single direction of causality. Finally, much of the apparent concordance between microvascular and macrovascular disease may reflect shared upstream drivers—diabetes, hypertension, obesity, dyslipidemia, and chronic inflammation—that independently promote injury in both compartments in parallel, rather than one compartment causing the other (81). Taken together, this evidence indicates that the microvascular-epicardial relationship is bidirectional, and that causal model restricted to a single direction is likely to be incomplete.

### 3.4 Prognostic implications of the microvascular-macrovascular link

The clinical significance of the microvascular-macrovascular link is underscored by robust prognostic data demonstrating that coronary microvascular dysfunction independently or in combination with macrovascular disease, predicts major adverse cardiovascular events, mortality, and progression to heart failure with preserved ejection fraction.

Impaired coronary flow reserve (CFR), a functional imaging marker of microvascular disease, is a powerful independent predictor of major adverse cardiovascular events (MACE) and mortality across diverse patient populations. A meta-analysis of 79 studies including nearly 60,000 individuals demonstrated that reduced CFR is associated with higher incidence of all-cause mortality and MACE, particularly in patients with isolated coronary microvascular dysfunction (82). Other studies have confirmed that impaired CFR is associated with poorer prognosis (83) and adverse cardiovascular events (84), with additional meta-analyses showing similar associations for PET-derived myocardial flow reserve (MFR) (85).

INOCA is now recognized as a high-risk clinical entity rather than a benign condition with increased rates of cardiovascular events, repeated hospital admissions, as well as impaired quality of life and increased healthcare costs (86, 87). Contemporary guidelines recommend assessment of coronary microvascular dysfunction in symptomatic patients with nonobstructive CAD, with stratified medical therapy guided by invasive coronary physiologic testing shown to improve angina severity and quality of life (88, 89). Schroder et al. in the iPOWER study of 1,681 women with angina and no obstructive CAD demonstrated that coronary flow velocity reserve (CFVR) was associated with composite outcome of cardiovascular death, MI, heart failure, stroke, and coronary revascularization (90). Toya et al. demonstrated that both CFR and hyperemic microvascular resistance as continuous variables predict future risk of MACE in patients with ischemia and nonobstructive coronary artery disease over median 8-year follow-up (91).

The relationship of microvascular disease (CMD) with heart failure with preserved ejection fraction (HFpEF) deserves special emphasis as it represents one of, if not the most important prognostic consequences of microvascular dysfunction. The multinational PROMIS-HFpEF study demonstrated that 75% of HFpEF patients have microvessel involvement (defined as CFR <2.5), and that microvascular disease was independently associated with cardiovascular and HF-related events at follow-up (92). The coexistence of both microvascular and diastolic dysfunction is associated with markedly increased risk of HFpEF hospitalization (93) and is linked to fibrosis, ventricular dysfunction, vascular rarefaction, and cardiovascular death (94, 95). Interestingly, a substantial proportion of INOCA patients meet criteria for HFpEF (96), underscoring the need for systematic evaluation of HFpEF in this population rather than treating INOCA and HFpEF as separate diagnostic considerations. SGLT2 inhibitors, which improve outcomes in HFpEF independently of diabetes status and have been proposed to improve microvascular and endothelial function, warrant further dedicated evaluation as a therapy specifically targeting the microvascular-HFpEF axis in patients with CMD, rather than being considered only after HFpEF is clinically overt.

Collectively, these data establish microvascular dysfunction as a critical determinant of prognosis across the spectrum of cardiovascular disease, both independently and in conjunction with epicardial atherosclerosis.

Table 3 summarizes the key prognostic studies demonstrating the clinical significance of microvascular dysfunction.

**Table 3:** Key prognostic studies demonstrating the clinical significance of microvascular dysfunction.

Study	Study design and population (*n*)	CMD assessment	Primary outcome (s)	Key findings	References
Kelshiker et al.	Meta-analysis (*n* = 59,740); coronary flow measures and events reported	CFR	All-cause mortality and MACE	Abnormal CFR: HR 3.78 for mortality, HR 3.42 for MACE; isolated CMD: HR 5.44 for mortality	(82)
Jensen et al.	Systematic review and meta-analysis (19 studies); No CAD with CMD	CFR	Death and MACE	Low CFR: HR 2.45 for death, HR 2.08 for MACE; similar in both sexes	(83)
Ahmed et al.	Meta-analysis (*n* = 46,815); prognostic value of MFR	MFR	Adverse CV outcomes	Impaired MFR: RR 2.94 for adverse outcomes	(85)
Green et al.	Meta-analysis (*n* = 11,867); prognostic value of CFR	CFR	MACE and hard CV events	Impaired CFR: HR 1.93 for MACE, HR 3.11 for hard events	(84)
iPOWER (Schroder et al.)	Prospective cohort; Women with angina and no obstructive CAD (*n* = 1,681)	CFVR	CV death, MI, HF, stroke, revascularization	HR 1.07 per 0.1 unit decrease in CFVR	(90)
Toya et al.	INOCA patients (*n* = 329); 8-year follow-up	CFR and HMR	MACE	Both CFR and HMR predicted MACE independent of CAD severity	(91)
PROMIS-HFpEF	Prospective study (*n* = 202); HfpEF patients	CFR	CV and HF-related events	75% of HfpEF had CMD; CMD independently associated with adverse events	(92)
Taqueti et al.	Retrospective cohort study (*n* = 201); suspected CAD with evaluation for CMD	CFR	HFpEF hospitalization	Combined CMD and diastolic dysfunction: >5-fold increased risk	(93)
Hong et al.	Prospective registry (*n* = 330); No LV systolic dysfunction and no significant stenosis	CFR	CV death or HF admission	Overt CMD: 43.8% vs. 14.5% CV death (diastolic vs. no diastolic dysfunction)	(95)

CMD, coronary microvascular disease; CFR, coronary flow reserve; MACE, major adverse cardiovascular events; HR, hazard ratio; CAD, coronary artery disease; MFR, myocardial flow reserve; CV, cardiovascular; RR, relative risk; CFVR, coronary flow velocity reserve; MI, myocardial infarction; HF, heart failure; INOCA, ischemia and no obstructive coronary artery disease; HMR, hyperemic microvascular resistance; HFpEF, heart failure with preserved ejection fraction; LV, left ventricular.

## 4 Sex differences and public health implications

Coronary microvascular disease represents a substantial and under-recognized public health burden, with contemporary population-based studies estimating that nearly half of individuals with angina have non-obstructive CAD (97), 60%–70% of which could be attributed to CMD (97), hence affecting millions of individuals in the United States alone. Importantly, noninvasive imaging markers of microvascular function, particularly PET- and MRI-derived CFR and MFR, consistently predict incident major cardiovascular events and mortality independent of obstructive CAD (82, 83, 85).

Sex-specific differences in the presentation and progression of ischemic heart disease further highlight the importance of microvascular pathology. Findings from the WISE program demonstrate that women are disproportionately affected by INOCA and CMD, likely reflecting differences in coronary anatomy, vascular biology, and susceptibility to endothelial dysfunction (98). Imaging studies indicate that in women, CMD and impaired perfusion frequently precede detectable epicardial atherosclerosis, contributing to a clinical phenotype characterized by persistent angina, diffuse ischemia, and progression to HFpEF (48, 99–102). Eventually, longstanding coronary CMD can reinforce epicardial atherosclerosis, particularly in patients with prolonged ischemia and comorbid risk factors.

In contrast, men usually develop obstructive epicardial plaques earlier than women, with infarction frequently being the first clinical manifestation (103, 104). However, microvascular dysfunction is not uncommon in men, with invasive physiology registries (105) demonstrating its presence even in the absence of obstructive CAD. Notably, impaired CFR confers a similarly elevated risk of adverse cardiovascular outcomes in both sexes (82) (106). While it is evident that phenotypic expression differs by sex, the full spectrum from microvascular to macrovascular disease exists in both sexes, but the timing, prevalence, and clinical consequences differ.

From an imaging perspective, these differences emphasize the need to move beyond a purely anatomical paradigm toward integrated functional and structural assessment of coronary disease. Reliance on lumen-based imaging alone may underestimate disease burden, particularly in populations in whom microvascular dysfunction predominates. In contrast, quantitative perfusion imaging and flow-based metrics enable earlier detection of disease, improved risk stratification, and more accurate phenotyping of ischemic heart disease.

The hypothesis that coronary microvascular dysfunction (CMD) may precede and promote atherogenesis has important public health and clinical implications. Earlier identification of CMD through quantitative perfusion or flow-reserve imaging with PET, CMR, or CT-derived techniques may detect disease at a stage when functional abnormalities precede overt structural atherosclerosis, creating an opportunity for earlier risk-factor modification and potentially reducing progression to clinical coronary disease. In patients with angina and/or objective ischemia without obstructive epicardial disease on angiography or CCTA, further evaluation for coronary vasomotor disorders may similarly enable identification of specific endotypes, including microvascular angina, vasospastic angina, or both. Invasive functional testing with coronary flow reserve, microvascular resistance assessment, and acetylcholine provocation can facilitate this distinction and guide endotype-specific therapy, such as calcium-channel blockers for vasospastic angina and therapies targeting microvascular dysfunction and endothelial function for microvascular angina. Beyond coronary disease, CMD is increasingly recognized as a central pathophysiological mechanism in HFpEF; impaired CFR is associated with substantially higher risks of hospitalization and adverse outcomes. Given the growing prevalence of HFpEF, incorporating assessment of CMD into the evaluation of patients with unexplained dyspnea or HFpEF—particularly those with ischemic symptoms or cardiometabolic risk factors—may therefore offer opportunities for earlier intervention and improved long-term outcomes. Collectively, a microvascular-centered paradigm could shift management from recognition of established epicardial disease toward earlier detection and modification of shared vascular dysfunction.

## 5 Summary and conclusion

This review supports an imaging-informed paradigm in which myocardial CMD may represent an upstream contributor to epicardial coronary atherogenesis within a bidirectional relationship between the microvascular and epicardial coronary compartments. Converging experimental, clinical, and imaging evidence suggests that microvascular dysfunction impairs coronary flow and distal runoff, leading to reduced wall shear stress (WSS) and endothelial dysfunction—key conditions that favor plaque initiation and progression.

Experimental models consistently demonstrate that microvascular abnormalities precede overt epicardial disease under cardiometabolic stress. In parallel, clinical imaging studies show that impaired myocardial perfusion and reduced coronary flow reserve frequently occur in the absence of obstructive coronary artery disease and are strongly associated with adverse outcomes. In combination with the well-described mismatch between perfusion defects and epicardial stenosis, together with the prognostic value of flow-based metrics, these observations offer support for a mechanistic link between microvascular dysfunction, ischemia, and atherogenesis.

These findings challenge a purely plaque-centric framework and highlight the limitations of anatomy-based assessment alone. Multimodality imaging—particularly PET, CMR, and CT perfusion—enables non-invasive quantitative evaluation of microvascular function and provides incremental diagnostic and prognostic value beyond luminal stenosis.

Recognizing CMD as an early and potentially modifiable component of coronary disease has important clinical and public health implications, particularly in cardiometabolic populations and in patients with ischemia and nonobstructive coronary arteries. A shift toward integrated structural and functional assessment may improve risk stratification and enable earlier, mechanism-based intervention.

Further studies are needed to define the temporal relationship between microvascular dysfunction and epicardial atherosclerosis and to determine whether targeting the microcirculation can alter disease progression and outcomes.

## 6 Limitations

The evidence reviewed spans experimental models, cross-sectional and prospective cohorts, multimodality imaging studies, meta-analyses, and expert consensus, which should not be considered equivalent in strength. Experimental and cross-sectional studies establish associations and, in animal models, temporal sequence, but cannot establish causality in humans. Prospective cohorts such as PESA provide stronger evidence for temporal precedence but remain observational and vulnerable to residual confounding from shared cardiometabolic risk factors, including diabetes, hypertension, obesity, dyslipidemia, and inflammation. The predominantly cross-sectional human literature also leaves open the possibility of reverse causation, whereby subclinical epicardial or diffuse vascular disease contributes to microvascular dysfunction. Furthermore, CMD assessment is heterogeneous, ranging from invasive measures such as coronary flow reserve and hyperemic microvascular resistance to PET- and CMR-derived myocardial flow reserve, limiting direct comparisons across studies. Animal models may also poorly reflect the slower, multifactorial course of human disease. Most importantly, no interventional trial has demonstrated that treating coronary microvascular dysfunction prevents the initiation or progression of epicardial atherosclerosis. Thus, the proposed pathway remains a hypothesis requiring prospective validation rather than an established therapeutic target.
